# Acupuncture Modulates the Spontaneous Activity and Functional Connectivity of Calcarine in Patients With Chronic Stable Angina Pectoris

**DOI:** 10.3389/fnmol.2022.842674

**Published:** 2022-04-26

**Authors:** Lei Lan, Tao Yin, Zilei Tian, Ying Lan, Ruirui Sun, Zhengjie Li, Miaomiao Jing, Qiao Wen, Shenghong Li, Fanrong Liang, Fang Zeng

**Affiliations:** ^1^Acupuncture and Tuina School, The 3rd Teaching Hospital, Chengdu University of Traditional Chinese Medicine, Chengdu, China; ^2^Acupuncture and Brain Science Research Center, Chengdu University of Traditional Chinese Medicine, Chengdu, China; ^3^Hospital of Chengdu University of Traditional Chinese Medicine, Chengdu, China; ^4^Gansu Provincial Hospital of Traditional Chinese Medicine, Lanzhou, China; ^5^State Key Laboratory of Southwestern Chinese Medicine Resources, Innovative Institute of Chinese Medicine and Pharmacy, Chengdu University of Traditional Chinese Medicine, Chengdu, China; ^6^Key Laboratory of Sichuan Province for Acupuncture and Chronobiology, Chengdu, China

**Keywords:** coronary artery disease, angina, brain-heart interaction, fractional amplitude of low-frequency fluctuations, functional connectivity, acupuncture

## Abstract

**Background:**

Acupuncture is an effective adjunctive therapy for chronic stable angina pectoris (CSAP), while the underlying mechanism is unclear. This study aimed to investigate the central pathophysiology of CSAP and explore the mechanism of different acupoint prescriptions for CSAP from the perspective of brain-heart interaction.

**Methods:**

Thirty-seven CSAP patients and sixty-five healthy subjects (HS) were enrolled, and thirty CSAP patients were divided into two acupoint prescriptions groups (Group A: acupoints on the meridian directly related to the *Heart*; Group B: acupoints on the meridian indirectly related to the *Heart*). The Magnetic Resonance Imaging data and clinical data were collected at baseline and after treatment. The comparisons of brain spontaneous activity patterns were performed between CSAP patients and HS, as well as between baseline and after treatment in CSAP patients. Then, the changes in resting-state functional connectivity before and after treatment were compared between the two acupoint prescriptions.

**Results:**

Chronic stable angina pectoris patients manifested higher spontaneous activity on the bilateral calcarine, left middle occipital gyrus, right superior temporal gyrus, and right postcentral gyrus. After acupuncture treatment, the spontaneous activity of the left calcarine, left cuneus, and right orbitofrontal gyrus was decreased. The left calcarine was identified as region-of-interest for functional connectivity analysis. Compared with group B, CSAP patients in group A had significantly increased functional connectivity between left calcarine and the left inferior temporal gyrus/cerebellum crus 1, left hippocampus, left thalamus, and left middle cingulate cortex after treatment. Thresholds for all comparisons were *p* < 0.05, Gaussian Random Field corrected.

**Conclusion:**

Regulating the aberrant spontaneous activity of the calcarine might be an underlying mechanism of acupuncture for CSAP. The multi-threaded modulation of functional connectivity between calcarine and multiple pain-related brain regions might be a potential mechanism for better efficacy of acupuncture at points on the meridian directly related to the *Heart*.

## Introduction

Chronic stable angina pectoris (CSAP) is a clinical syndrome characterized by constricting discomfort in the chest, jaw, shoulder, or back, typically aggravated by exertion or emotional stress (Piccolo et al., 2015). As the most common manifestation of coronary artery disease (CAD; Benjamin et al., 2017), CSAP has been identified as a predominant risk of major cardiovascular events and sudden cardiac death, and brings a significant impact on functional capacity and quality of life of patients (Chaitman and Laddu, 2011). Therefore, searching for the effective management of CSAP to reduce the frequency of angina attacks is of great value. Acupuncture is a widely used traditional practice for the prevention and treatment of angina and has been identified as a safe and effective adjunctive therapy for CSAP in several high-quality clinical trials (Zhao et al., 2019, 2021; Huang et al., 2021). It was reported that acupuncture could effectively alleviate symptoms, reduce the frequency of angina attacks, and decrease nitroglycerin use in CSAP patients (Shen et al., 2021). However, the underlying mechanism of acupuncture for CSAP was still unclear.

Recently, the proposal of brain-heart interaction theory (Silvani et al., 2016) provides a new perspective to explore the pathogenesis of cardiovascular diseases and explain the mechanism of intervention. For example, evidence from functional Magnetic Resonance Imaging (fMRI) demonstrated that the dysfunction of autonomic-limbic integration might be the important pathophysiology of takotsubo syndrome (Templin et al., 2019), and the hyperactivity of the temporal gyrus, parahippocampus, fusiform gyrus, and cerebellum played a critical role in pain perception response in CAD patients (Wittbrodt et al., 2020). Furthermore, during the dobutamine-induced angina task, patients with angina manifested increased regional cerebral blood flow in the thalamus, periaqueductal gray, prefrontal cortex, and anterior cingulate cortex (Rosen et al., 1994). These regions overlapped highly with the findings of previous neuroimaging studies about pain (Davis et al., 2017; Mouraux and Iannetti, 2018), which suggested that angina pain, a typical kind of visceral nociception, might share the similarity in pain processing with somatalgia. As an increasing number of studies have detected that acupuncture could modulate the activities of pain processing-related regions, including the thalamus (Chu et al., 2012), hippocampus (Ma et al., 2020), medulla oblongata-brainstem (Chang et al., 2021), middle temporal gyrus (Yu et al., 2019), and occipital gyrus (Ma et al., 2020) in patients with chronic pain, it was necessary and feasible to explore the mechanism of acupuncture for treating CSAP by functional neuroimaging techniques.

Therefore, this resting-state fMRI study was conducted, with the following three aims: (1) comparing the brain spontaneous activity patterns between CSAP patients and healthy subjects (HS), and (2) investigating how acupuncture modulates the abnormal activity pattern of CSAP patients, and (3) exploring the differences of two acupoint prescriptions on regulating resting-state functional connectivity (rsFC) of CSAP patients. We hypothesized that CSAP patients had aberrant functional activity in these brain regions closely associated with pain processing, which could be significantly modulated by acupuncture treatment, and that different acupoint prescriptions had different regulating effects on rsFC of CSAP patients.

## Materials and Methods

### Study Design

**FIGURE 1:**
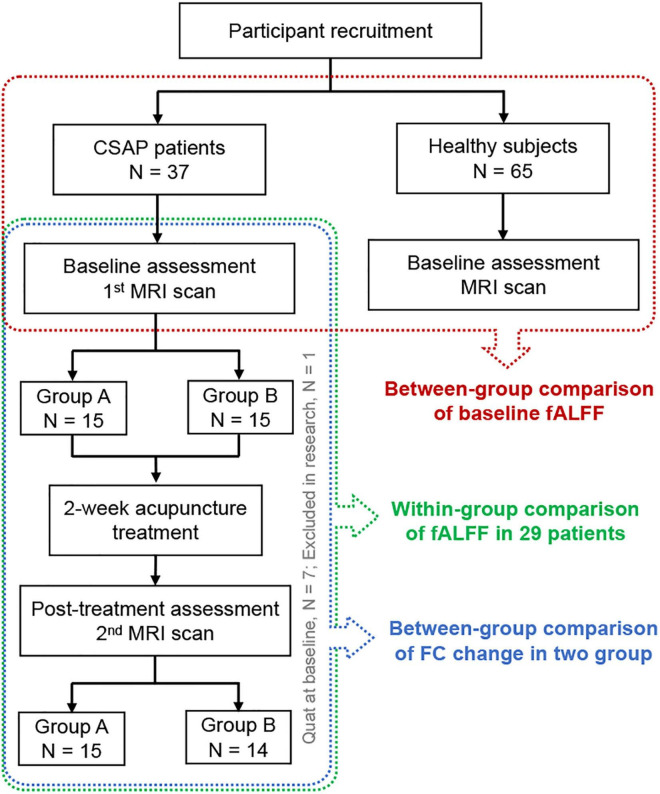
The flowchart of the study. CSAP, chronic stable angina pectoris; MRI, Magnetic Resonance Imaging; fALFF, fractional Amplitude of Low-Frequency Fluctuation; FC, functional connectivity.

This trial consisted of a 2-week baseline phase and a 2-week treatment phase. CSAP patients underwent clinical evaluation and MRI scans at the baseline and end of the treatment phase. HS underwent evaluation and MRI scans at the baseline. The flowchart of the study is shown in Figure 1.

### Ethical Approval and Trial Registration

This study was approved by the Sichuan Traditional Chinese Medicine Regional Ethics Committee (No. 2013KL-14) and was registered on ChiCTR (No. ChiCTR-TRC-13003265). The study was conducted in accordance with the Declaration of Helsinki. All the participants provided written informed consent.

### Participants

A total of 37 CSAP patients and 65 healthy subjects (HS) were enrolled in this study. The patients were recruited from the Third Teaching Hospital of Chengdu University of Traditional Chinese Medicine and Sichuan Second Hospital of Traditional Chinese Medicine. HS came from the nearby communities.

The diagnostic criteria of CSAP followed the guidelines for the management of patients with chronic stable angina of the American College of Cardiology/American Heart Association (Fraker et al., 2007) and the Chinese Society of Cardiology (Chinese Society of Cardiology, Chinese Medical Association, and Editorial Board of Chinese Journal of Cardiology, 2007). Patients were included if they fulfilled all of the following items: (1) aged from 45 to 80 years old; (2) right-handedness; (3) met the diagnostic criteria; (4) graded as level 1 or 2 of angina based on the angina grading system of the Canadian Cardiovascular Society (Smith, 2002); (5) coronary artery stenosis ≥50% on coronary angiography; (6) the duration of disease was longer than 3 months; (7) the frequency of angina attacks was more than twice a week. The patients were excluded if they matched one of the following items: (1) had an acute coronary syndrome, malignant arrhythmia, or myocardial infarction within the last 3 months; (2) suffered from other severe psychiatric, neurological, cardiovascular, respiratory, or renal disorders; (3) had a diagnosis of diabetes or impaired glucose tolerance; (4) suffered from other chronic pain disorders or having a history of head trauma; (5) had any contraindication to MRI scanning, such as claustrophobia; (6) the Zung Self-Rating Anxiety Scale (SAS) or Self-Rating Depression Scale (SDS) ≥50; (7) participated in other clinical trials within 3 months; (8) received acupuncture treatment in the past 3 months.

Healthy subjects should meet the following criteria for inclusion: (1) aged from 45 to 80 years old; (2) right-handedness; (3) had no CSAP or other physical or psychological diseases. The exclusion criteria of HS were as follows: (1) had any contraindication to MRI scanning, such as claustrophobia; (2) participated in other clinical trials within 3 months; (3) received acupuncture treatment in the past 3 months.

All participants underwent clinical assessment, physical examination, and laboratory tests after recruitment. The cardiologists from Chengdu University of Traditional Chinese Medicine determined whether a participant could be included based on the inclusion criteria and physical examination results.

### Intervention

Patients willing to receive acupuncture treatment were assigned into two groups randomly using a random number table. The acupoint prescriptions were as follows: Group A (acupoints on the meridian directly related to the *Heart*): bilateral *Neiguan* (PC6) and bilateral *Tongli* (HT5); Group B (acupoints on the meridian indirectly related to the *Heart*): bilateral *Yangxi* (LI5) and bilateral *Pianli* (LI6). The location of these acupoints is provided in Supplementary Figure 1. All acupoints were stimulated with the 1.5 *cun* (diameter 0.25 mm, length 40 mm) filiform acupuncture needles. One licensed acupuncturist with more than 5 years of clinical experience administered all the acupuncture manipulation. First, the needles were inserted into the acupoints 5–15 mm perpendicularly. Then the acupuncturist lifted and thrusted the needles with an amplitude of 3–5 mm, twirled the needles in 90–180° to achieve *deqi* sensation. The needles were retained in the acupoints for 30 min, during which the needles were manipulated every 10 min for 2 times, with each time taking 10–15 s.

The treatment sessions lasted for 2 weeks, with five consecutive days per week followed by 2 days off for a total of 10 acupuncture treatments. During the treatment, the regular use of aspirin, clopidogrel, beta-adrenergic blocking agents, statins, angiotensin-converting enzyme inhibitors were allowed as basic management for all CSAP patients (Fraker et al., 2007). HS received no acupuncture treatment.

### Outcome Measurements

The primary outcome was the frequency of angina attacks for 2 weeks, and the secondary outcomes included the McGill pain score, SAS, and SDS. The McGill Pain Questionnaire is a personalized measurement tool for pain experience (Melzack, 1975), which could be used to monitor the pain intensity and affectivity, and to determine the effectiveness of interventions. The SAS and SDS are self-reported scales for evaluating patients’ anxiety and depression status.

### Statistical Analysis

All the clinical data were analyzed with SPSS 20 software (IBM, Armonk, NY, United States). Shapiro–Wilk test was used to evaluate the normality of the data. The comparisons between the demographic characteristics of CSAP patients and HS, as well as between symptom improvements of Group A and Group B were performed with the two-sample *t*-tests or *Chi-square* tests. The comparisons of clinical outcomes within the pre- and post-treatment were conducted with the paired *t*-test. All the statistical thresholds were set at *p* < 0.05, two-tailed.

### Magnetic Resonance Imaging Scans

Magnetic resonance imaging data were collected using a 3.0T MRI scanner (Siemens, Munich, Germany) with an eight-channel phased-array head coil at the West China Hospital of Sichuan University. Each scanning session included a T1-weighted imaging scan and a resting-state blood oxygen level-dependent (BOLD) imaging scan.

Participants were required to stay awake and keep their heads still during the scan, with their eyes closed and ears plugged. The T1 image were obtained using a fast spoiled gradient recalled sequence (slice thickness = 1 mm, repetition time = 2700 ms, echo time = 3.39 ms, field of view = 256 mm, flip angle = 7°, matrix = 256 × 256). The BOLD image was obtained using the echo-planar imaging (slice number = 30, total volumes: 180, slice thickness = 5 mm, repetition time = 2000 ms, echo time = 30 ms, field of view = 240 mm, flip angle = 90°, matrix = 64 × 64).

### Magnetic Resonance Imaging Data Processing

#### Data Preprocessing

Magnetic resonance imaging data were preprocessed with the DPABI toolbox (Yan et al., 2016)^1^. The data preprocessing included the following steps: (1) discarding of the first ten time points; (2) slice timing correction; (3) realignment and head motion correction; (4) excluding participants with excessive head motion [mean framewise displacement (Jenkinson et al., 2002) >0.2]; (5) spatial normalization into Montreal Neurological Institute space through Diffeomorphic Anatomical Registration Through Exponentiated Lie Algebra (Ashburner, 2007); (6) spatial smoothing with 4 mm full width half maximum Gaussian; (7) regression of confounding factors (white matter, cerebrospinal fluid, and linear and quadratic trends); (8) temporal filtering (0.01–0.1 Hz) of the time series was performed after the fractional Amplitude of Low-Frequency Fluctuation (fALFF) analysis.

#### Fractional Amplitude of Low-Frequency Fluctuation Analysis

After checking the quality of images, the whole-brain fALFF was calculated for every participant. In the first part of analysis, a two-sample *t*-test was performed to compare the between-group difference of the baseline fALFF in CSAP patients and HS, with the age, gender, education level, Body Mass Index (BMI), and mean framewise displacement as covariances. In the second part of analysis, a paired *t*-test was conducted between the pre- and post-treatment fALFF of all CSAP patients to investigate the modulating effects of acupuncture. The threshold of these comparisons was set to *p* < 0.05 (two-tailed) at both voxel level and cluster level, corrected with the Gaussian Random Field (GRF) method.

Subsequently, the results of the first and second part of analysis were overlapped, to extract the key region that participates both in the neuropathology and acupuncture treatment effects of CSAP. Then, the partial correlation analyses between the baseline fALFF of the overlapping region and the baseline clinical symptoms, as well as between the fALFF change of this region and the clinical measures improvements after treatment were performed in CSAP patients, with age, gender, education level, BMI, and head motion as covariates. The statistical threshold for correlation analyses was set at *p* < 0.05.

#### Resting-State Functional Connectivity Analysis

The overlapping region identified above was set as region-of-interest (ROI), and the ROI-to-voxel rsFC maps were calculated for all patients. To investigate the specific effects of different acupoint prescriptions for the rsFC of CSAP patients, the within-group comparisons between the pre- and post-treatment rsFC maps in Group A and Group B, as well as the between-group comparison of the rsFC maps change between Group A and B were performed. The within-group comparisons were conducted with the paired *t*-test. The between-group comparison was performed with the two-sample *t*-test, using age, gender, education level, and BMI as covariates. The threshold of these comparisons was set to voxel-level *p* < 0.05 and cluster-level *p-GRF* < 0.05, two-tailed. Furthermore, the Spearman correlation analyses between rsFC change and clinical measures improvements after acupuncture treatment in Group A, Group B, and all the patients were also performed for the non-normal distribution of clinical data in both group A and B. The statistical threshold for correlation analyses was set at *p* < 0.05.

## Results

### Demographic and Clinical Characteristics of Chronic Stable Angina Pectoris Patients and Healthy Subjects

In total, 37 CSAP patients and 65 HS were included. The age of CSAP patients was higher than HS. There was no statistical difference in gender, BMI, and education level between CSAP patients and HS (Table 1). No statistical difference in mean framewise displacement was found between CSAP patients (0.12 ± 0.06) and HS (0.10 ± 0.05). And there were no significant pharmacological intervention differences or baseline characteristic differences between Group A and Group B (Table 2).

**TABLE 1:** The demographic and clinical characteristics of CSAP patients and HS.

	CSAP (*n* = 37) (mean ± SD)	HS (*n* = 65) (mean ± SD)	Statistic value	*p* value
Age (Years)	65.05 ± 7.23	56.71 ± 5.49	*t* = 6.092	<0.001***
Gender (M/F)	20/17	26/39	*x*^2^ = 0.342	0.559
BMI (Kg/m^2^)	24.42 ± 2.67	23.35 ± 2.72	*t* = 1.911	0.059
Education level (primary/middle school/college)	6/22/9	8/40/17	*x*^2^ = 0.310	0.856
Duration (Month)	57.92 ± 56.78	/	/	/
Frequency of angina attacks	5.89 ± 4.38	/	/	/
McGill pain scale	10.46 ± 3.82	/	/	/
SAS score	32.97 ± 4.51	/	/	/
SDS score	30.95 ± 4.70	/	/	/

CSAP, chronic stable angina pectoris; HS, healthy subjects; M/F, male/female; BMI, Body Mass Index; SAS, self-rating anxiety scale; SDS, self-rating depression scale. ***p < 0.001.

**TABLE 2:** The between-group comparison of demographic characteristics and baseline conditions of CSAP patients in these two acupuncture groups.

	Group A (*n* = 15) (mean ± SD)	Group B (*n* = 14) (mean ± SD)	Statistic value	*p* value
Age (Years)	65.20 ± 5.967	65.86 ± 7.675	*t* = −0.258	0.189
Gender (M/F)	6/9	8/6	*x*^2^ = 0.852	0.356
BMI (Kg/m^2^)	25.05 ± 2.94	24.56 ± 2.56	*t* = 0.478	0.593
Education level (primary/middle school/college)	3/10/2	2/8/4	*x*^2^ = 1.056	0.590
Duration (Month)	62.33 ± 52.64	59.79 ± 71.46	*t* = 0.110	0.913
Frequency of angina attacks	5.53 ± 3.60	5.43 ± 4.85	*t* = 0.689	0.414
McGill pain score	10.73 ± 4.76	9.57 ± 2.64	*t* = 0.363	0.552
SAS score	33.58 ± 3.83	32.68 ± 5.32	*t* = 0.024	0.877
SDS score	31.93 ± 5.02	30.36 ± 5.08	*t* = 0.211	0.650
**Drug use**				
Antiplatelet drugs (Y/N)	10/5	8/6	*x*^2^ = 0.279	0.597
ACEI/ARB (Y/N)	4/11	3/11	*x*^2^ = 0.109	0.742
Beta-adrenergic blocking agents (Y/N)	5/10	6/8	*x*^2^ = 0.279	0.597
Statins	11/4	9/5	*x*^2^ = 0.277	0.599

*M/F, male/female; BMI, Body Mass Index; SAS, self-rating anxiety scale; SDS, self-rating depression scale; Y/N, Yes/No; ACEI, angiotensin-converting enzyme inhibitors; ARB, angiotensin receptor Blocker.*

### Baseline Comparison of the Fractional Amplitude of Low-Frequency Fluctuation Between Chronic Stable Angina Pectoris Patients and Healthy Subjects

**FIGURE 2:**
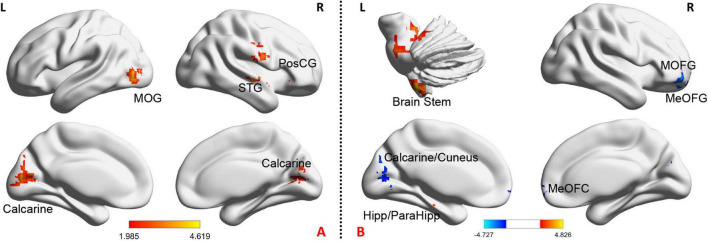
The baseline and the change of fALFF after treatment in CSAP patients. **(A)** Illustrates the baseline comparison of fALFF between CSAP patients and HS. The warm tone indicates CSAP patients >HS. **(B)** Illustrates acupuncture effects on the fALFF of CSAP patients. The warm tone indicates increased fALFF after treatment while the cool tone indicates decreased fALFF after treatment. Threshold: voxel-level *p* < 0.05, cluster-level *p-GRF* < 0.05. MOG, middle occipital gyrus; PosCG, postcentral gyrus; STG, superior temporal gyrus; MOFG, middle orbitofrontal gyrus; MeOFG, median orbitofrontal gyrus; ParaHipp, parahippocampus; Hipp, hippocampus; L, left; R, right.

Chronic stable angina pectoris patients had significantly higher fALFF in the bilateral calcarine, left middle occipital gyrus (MOG), right superior temporal gyrus, and right postcentral gyrus, while no region with lower fALFF than HS (voxel-level *p* < 0.05, cluster-level *p-GRF* < 0.05) (Figure 2A and Supplementary Table 1).

### Acupuncture Effects on the Clinical Symptoms of Chronic Stable Angina Pectoris Patients

Seven CSAP patients were unwilling to receive acupuncture treatment and quit at the baseline. One CSAP patient was excluded due to the excessive head motion in the second scan. Therefore, 29 CSAP patients (15 patients in group A and 14 patients in group B) with eligible clinical and imaging data were retained for the following analysis.

The within-group analysis among these 29 patients demonstrated that acupuncture treatment could significantly improve the McGill pain score (*p* < 0.001) and SAS score (*p* < 0.05). Patients in Group A had significant improvements in the frequency of angina attacks (*p* < 0.05), McGill pain scale (*p* < 0.01), and SAS score (*p* < 0.05), while patients in Group B only had a significant improvement in McGill pain scale (*p* < 0.001) after acupuncture treatment. There was no significant between-group difference in these 4 metrics in Group A and Group B (*p* > 0.05) (Table 3).

**TABLE 3:** The clinical effects of acupuncture in CSAP patients.

	Pre (mean ± SD)	Pos (mean ± SD)	Within-group comparison		Between-group comparison [(A_pos –_ A_pre_) vs. (B_pos_ – B_pre_)]	
			*t* value	*p* value	*t* value	*p* value
**Frequency of angina attacks**						
All patients	5.48 ± 4.17	4.45 ± 3.73	1.240	0.225		
Group A (*n* = 15)	5.53 ± 3.60	3.93 ± 3.26	1.964	0.049*	0.689	0.414
Group B (*n* = 14)	5.43 ± 4.85	5.00 ± 4.22	0.528	0.598		
**McGill pain score**						
All patients	10.17 ± 3.86	6.36 ± 2.95	6.101	<0.001***		
Group A (*n* = 15)	10.73 ± 4.76	6.87 ± 2.61	3.665	0.003**	0.363	0.552
Group B (*n* = 14)	9.57 ± 2.64	5.82 ± 3.28	5.557	<0.001***		
**SAS score**						
All patients	33.15 ± 4.55	31.19 ± 4.55	2.328	0.027*		
Group A (*n* = 15)	33.58 ± 3.83	31.29 ± 4.64	2.427	0.029*	0.024	0.877
Group B (*n* = 14)	32.68 ± 5.32	31.07 ± 4.62	1.104	0.290		
**SDS score**						
All patients	31.17 ± 5.02	30.52 ± 5.13	0.674	0.506		
Group A (*n* = 15)	31.93 ± 5.02	30.50 ± 5.51	1.264	0.227	0.211	0.650
Group B (*n* = 14)	30.36 ± 5.08	30.54 ± 4.90	−0.110	0.914		

Pre, Pre-treatment; Pos, Pos-treatment; SD, standard deviation; SAS, self-rating anxiety scale; SDS, self-rating depression scale. *p < 0.05; **p < 0.01; ***p < 0.001.

### Acupuncture Effects on the Fractional Amplitude of Low-Frequency Fluctuation of Chronic Stable Angina Pectoris Patients

After acupuncture treatment, CSAP patients manifested significantly increased fALFF in the brain stem, hippocampus, and parahippocampus, as well as decreased fALFF in the left calcarine, left cuneus, right middle orbitofrontal gyrus, and right median orbitofrontal gyrus (voxel-level *p* < 0.05, cluster-level *p-GRF* < 0.05) (Figure 2B and Supplementary Table 2). In addition, Supplementary Figure 2 and Supplementary Table 3 displayed the between-group differences of fALFF changes in these two groups.

### Regulation of Acupuncture on the Aberrant Fractional Amplitude of Low-Frequency Fluctuation in Chronic Stable Angina Pectoris Patients

**FIGURE 3:**
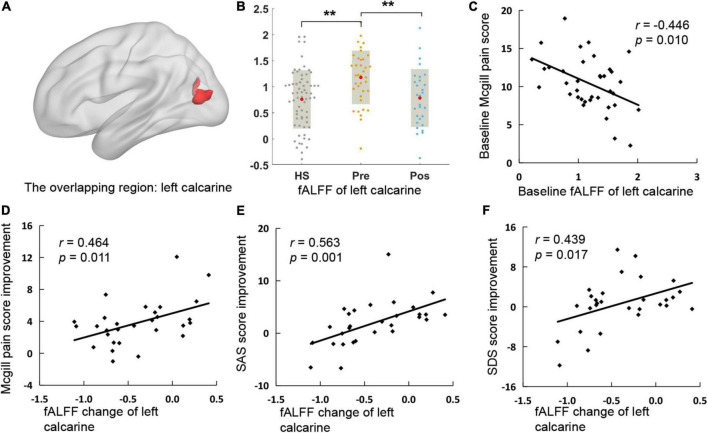
The overlapping region and its correlation with clinical symptoms. **(A)** Is the location of the overlapping region. **(B)** Is the comparison of fALFF of the overlapping region among HS and the baseline and after treatment of CSAP patients. **(C)** Illustrates the correlation between the baseline fALFF of the overlapping region and the baseline McGill pain score in CSAP patients. **(D–F)** Illustrate the correlations between the fALFF change of the overlapping region and improvements of clinical symptoms after treatment. HS, healthy subjects; pre, pre-treatment; pos, post-treatment; fALFF, fractional Amplitude of Low-Frequency Fluctuation; SAS, self-rating anxiety scale; SDS, self-rating depression scale. *p < 0.05; **p < 0.01; ***p < 0.001.

This study further performed an overlapping analysis between regions that survived in the baseline comparison and regions that changed after treatment in CSAP patients, finding that the overlapping region was located at the left calcarine (cluster size = 41) (Figure 3A). There were significant differences in fALFF of this region between CSAP patients and HS, as well as between the baseline and after treatment in CSAP patients (Figure 3B). In addition, the baseline fALFF of this region was significantly correlated with the baseline McGill pain score of CSAP patients (*r* = −0.446, *p* = 0.010) (Figure 3C). The change in fALFF of this region was significantly correlated with the improvement of McGill pain score (*r* = 0.464, *p* = 0.011), SAS score (*r* = 0.563, *p* = 0.001), and SDS score (*r* = 0.439, *p* = 0.017) in CSAP patients (Figures 3D–F).

### Variation of Different Prescriptions on the Modulation of Resting-State Functional Connectivity in Chronic Stable Angina Pectoris Patients

**FIGURE 4:**
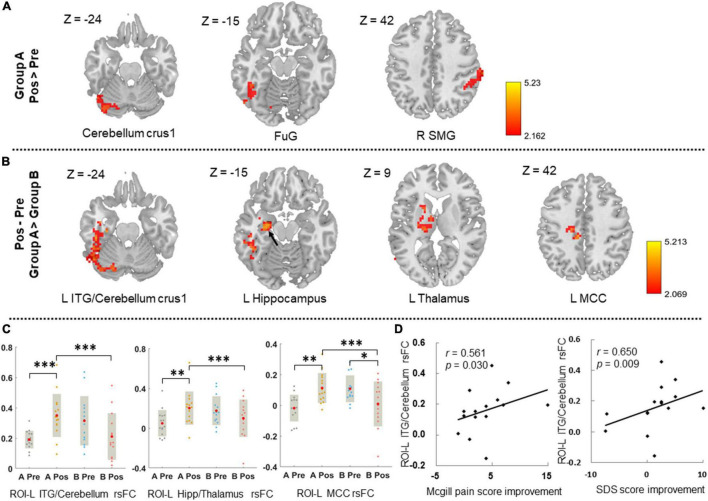
The ROI-based rsFC change after treatment and its correlations with clinical symptoms improvements. **(A)** Displays the ROI-based rsFC change after treatment in group A. Threshold: voxel-level *p* < 0.05, cluster-level *p-GRF* < 0.05. **(B)** Displays the between-group difference of ROI-based rsFC change after treatment in groups A and B. Threshold: voxel-level *p* < 0.05, cluster-level *p-GRF* < 0.05. **(C)** Illustrates the comparisons of ROI-based rsFC values of the survival regions at pre- and post-treatment in those two groups. **(D)** Illustrates the correlations between ROI-based rsFC change of the survival regions and clinical symptoms improvements in group A. L, left; R, right; FuG, fusiform gyrus; SMG, supramarginal gyrus; ITG, inferior temporal gyrus; MCC, middle cingulate cortex; Hipp, hippocampus; ROI, region-of-interest; rsFC, resting-state functional connectivity; SDS, self-rating depression scale; *p < 0.05; ^**^p < 0.01; ^***^p < 0.001. The black arrow indicates the location of the left hippocampus.

The overlapping region (the left calcarine) was set as the ROI for the ROI-voxel rsFC analysis. Patients in Group A manifested increased rsFC between ROI and the left cerebellum crus 1, left fusiform gyrus, and right supramarginal gyrus, while no significant rsFC change was found in Group B after treatment (Figure 4A and Supplementary Table 4).

Compared to group B, CSAP patients in group A had significantly increased rsFC between ROI and the left inferior temporal gyrus (ITG)/cerebellum crus 1, left hippocampus/thalamus, and left middle cingulate cortex (MCC) after acupuncture treatment (voxel-level *p* < 0.05, cluster-level *p-GRF* < 0.05) (Figures 4B,C and Supplementary Table 5).

The rsFC change between the ROI and left ITG/cerebellum crus 1 was significantly correlated with McGill pain score improvement (*r* = 0.561, *p* = 0.030) and SDS score improvement in Group A (*r* = 0.650, *p* = 0.009) (Figure 4D), while no significant correlation in group B or in all 29 patients.

## Discussion

To the best of our knowledge, this was the first study to investigate the aberrant brain spontaneous activity patterns of CSAP patients and explore the modulating effects of acupuncture for functional brain activity as well as rsFC in CSAP patients. The findings demonstrated that acupuncture could significantly decrease the elevated spontaneous activity of the left calcarine in CSAP patients. Compared with the acupoints on the meridian indirectly related to the *Heart*, acupuncturing at the points on the meridians directly related to the *Heart* had a remarkable effect on the regulation of rsFC between the calcarine and the ITG, cerebellum crus 1, hippocampus, thalamus, and MCC.

Brain-heart interaction plays an important role in the pathophysiology and treatment of cardiovascular diseases (Silvani et al., 2016). On the one hand, the heart receives signals from the brain via the sympathetic and parasympathetic nerves which are controlled by the central autonomic network (Benarroch, 1993). On the other hand, the noxious pain stimulus of angina is mediated by coronary chemoreceptors and transmitted to the brain via vagal afferent nerves, and finally integrated and processed in the cerebral cortex. The current study demonstrated that CSAP patients manifested higher spontaneous activity on the calcarine, MOG, and postcentral gyrus, and that the abnormally elevated functional activity of the calcarine was positively correlated with the McGill pain score in patients. The postcentral gyrus is the cortex center of somatic sensation perception and the crucial part of the pain neural matrix, which is responsible for the adjustment of pain perception, including the positioning and recognition of pain intensity (Mouraux and Iannetti, 2018). Calcarine and MOG are important components of the primary visual cortex and are traditionally thought to be primarily involved in visual processing (Thiebaut de Schotten et al., 2014). However, there is growing evidence in recent years found that several components of the visual cortex, including the calcarine and MOG, were also involved in the processing of pain signals. Results of several fMRI studies have indicated that patients with chronic pain, such as migraine (Wei et al., 2019), low back pain (Bush et al., 2021), and persistent somatoform pain disorder (Liu et al., 2019), exhibited atypical functional activity patterns in the occipital gyrus. For example, (Wei et al., 2019) demonstrated that migraine patients had aberrant spontaneous activity and regional homogeneity in calcarine, as well as atypical rsFC between calcarine and the thalamus, the relay station of sensory signaling. Moreover, these abnormalities of calcarine activity and connectivity patterns were significantly correlated with the pain intensity and affective condition of patients (Wei et al., 2019, 2021). This evidence indicated that calcarine was involved in the processing of pain perception and pain affectivity (Schwedt et al., 2015). In addition to the somatalgia, patients with chronic visceral pain (e.g., irritable bowel syndrome) also exhibited higher spontaneous activity and regional homogeneity in calcarine at resting-state (Chen et al., 2021), and increased activity in the visual cortex during the expectation of rectal pain. These findings suggested that the calcarine was not only associated with pain perception and pain affectivity but was also involved in hypervigilance to pain anticipation (Lee et al., 2012). The typical symptom of CSAP is prolonged, episodic chest dullness and crushing pain. This noxious pain stimulation as well as alertness and excessive fear of angina attacks lead to the hypersensitization of calcarine, manifested as symptoms-associated hyperactivity of the calcarine in CSAP patients.

After acupuncture treatment, the pain experience and anxiety condition were significantly improved, the abnormally elevated functional activity of the calcarine was significantly normalized in CSAP patients, and there were positive correlations between fALFF change in calcarine and improvements of McGill pain, SAS, and SDS score in patients. These findings reverified that acupuncture was an effective adjunctive therapy for CSAP, which was consisted with the previous multicenter randomized controlled trial (Zhao et al., 2019), and suggested that the effects of acupuncture in improving the pain experience of CSAP patients were closely related to the modulation of calcarine activity. In addition, this study also found that acupuncture treatment could significantly regulate the spontaneous activity of the brainstem, hippocampus, parahippocampus, and orbitofrontal cortex in CSAP patients, which were closely related to the transmission, perception, attention, and cognition of pain (Bushnell et al., 2013). These findings indicated that acupuncture for analgesia was not single-targeted. The improvements of symptoms in CSAP patients induced by acupuncture were associated with its multi-targeted modulations of the pain process. These similar results have also been observed in acupuncture treatment of other visceral pains and somatalgia (Ma et al., 2020, 2021; Wen et al., 2021).

Another important finding of this study was the difference in clinical efficacy and modulation of rsFC patterns between acupuncture at points on the meridian indirectly and directly related to the *Heart*. These results supported the classic acupuncture theory “selecting acupoints along the meridian” (Xing et al., 2013) and explained the potential mechanism of better effects of acupoints on the meridian directly related to the *Heart* for CSAP from the perspective of brain-heart interaction. This study compared the differences of ROI-voxel rsFC change between these two acupoint prescriptions, finding that acupuncturing at points on the meridian directly related to the *Heart* could significantly increase the rsFC between the calcarine and the thalamus, hippocampus, MCC, and ITG/Cerebellum crus 1 than acupoints on the meridian indirectly related to the *Heart*. Pain is a multidimensional and complex experience involving sensory, cognitive, and affective aspects (Fitzcharles et al., 2021). The thalamus is a pivotal node for pain transmission and mediation, the hippocampus and cingulate cortex are responsible for the processing and encoding of pain cognition and affectivity, while the cerebellum and ITG play an important role in pain visual perception and multimodal sensory integration (Saab, 2012; Bushnell et al., 2013; Finnerup et al., 2021). Therefore, the current findings, while confirming the specificity of the acupoint effects, also suggested that better improvement of CSAP symptoms by acupuncturing at points on the meridian directly related to the *Heart* might be correlated with their multi-threaded modulations to the pain network.

Several limitations should be concerned in this study. First, the sample size was smaller in each acupuncture treatment group. Second, the intervention phase was short. These two factors may account for the non-significant difference in clinical efficacy between two acupoint prescriptions. Third, to be consistent with clinical practice, patients in this study are allowed to receive recommended pharmacological interventions. Therefore, the effects of drugs on functional brain activity are difficult to exclude.

## Conclusion

The current study provided neuroimaging evidence for understanding the central pathophysiology of CSAP patients and the mechanism of acupuncture for CSAP. These findings suggested that the elevated spontaneous activity of the calcarine was an important central pathological characteristic of CSAP, and regulation of the aberrant spontaneous activity of the calcarine might be an underlying mechanism of acupuncture treatment for CSAP patients. The multi-threaded modulation of rsFC between calcarine and multiple pain-related brain regions might be a potential mechanism for better efficacy of acupuncture at points on the meridian directly related to the *Heart*.

## Data Availability Statement

The raw data supporting the conclusions of this article will be made available by the authors, without undue reservation.

## Ethics Statement

The studies involving human participants were reviewed and approved by the Sichuan Traditional Chinese Medicine Regional Ethics Committee. The patients/participants provided their written informed consent to participate in this study.

## Author Contributions

FL and FZ conceived and designed the study. YL, RS, ZL, MJ, and QW recruited the participants. TY and ZT analyzed the data. LL drafted the manuscript. SL, FL, and FZ revised the manuscript. All authors contributed to the article and approved the submitted version.

## Conflict of Interest

The authors declare that the research was conducted in the absence of any commercial or financial relationships that could be construed as a potential conflict of interest.

## Publisher’s Note

All claims expressed in this article are solely those of the authors and do not necessarily represent those of their affiliated organizations, or those of the publisher, the editors and the reviewers. Any product that may be evaluated in this article, or claim that may be made by its manufacturer, is not guaranteed or endorsed by the publisher.

## Footnotes

1
http://rfmri.org/dpabi

## References

[B1] Ashburner J. (2007). A fast diffeomorphic image registration algorithm. *Neuroimage* 38 95–113. 10.1016/j.neuroimage.2007.07.007 17761438

[B2] Benarroch E. E. (1993). The central autonomic network: functional organization, dysfunction, and perspective. *Mayo Clin. Proc.* 68 988–1001. 10.1016/s0025-6196(12)62272-1 8412366

[B3] Benjamin E. J. Blaha M. J. Chiuve S. E. Cushman M. Das S. R. Deo R. (2017). Heart Disease and Stroke Statistics-2017 Update: a Report From the American Heart Association. *Circulation* 135 e146–e603. 10.1161/cir.0000000000000485 28122885 PMC5408160

[B4] Bush N. J. Schneider V. Sevel L. Bishop M. D. Boissoneault J. (2021). Associations of Regional and Network Functional Connectivity With Exercise-Induced Low Back Pain. *J. Pain* 22 1606–1616. 10.1016/j.jpain.2021.05.004 34111507 PMC9527042

[B5] Bushnell M. C. Ceko M. Low L. A. (2013). Cognitive and emotional control of pain and its disruption in chronic pain. *Nat. Rev. Neurosci.* 14 502–511. 10.1038/nrn3516 23719569 PMC4465351

[B6] Chaitman B. R. Laddu A. A. (2011). Stable angina pectoris: antianginal therapies and future directions. *Nat. Rev. Cardiol.* 9 40–52. 10.1038/nrcardio.2011.129 21878880

[B7] Chang C. M. Yang C. P. Yang C. C. Shih P. H. Wang S. J. (2021). Evidence of Potential Mechanisms of Acupuncture from Functional MRI Data for Migraine Prophylaxis. *Curr. Pain Headache Rep.* 25:49. 10.1007/s11916-021-00961-4 34036477

[B8] Chen X. F. Guo Y. Lu X. Q. Qi L. Xu K. H. Chen Y. (2021). Aberrant Intraregional Brain Activity and Functional Connectivity in Patients With Diarrhea-Predominant Irritable Bowel Syndrome. *Front. Neurosci.* 15:721822. 10.3389/fnins.2021.721822 34539337 PMC8446353

[B9] Chinese Society of Cardiology, Chinese Medical Association, and Editorial Board of Chinese Journal of Cardiology (2007). Guideline for diagnosis and treatment of patients with chronic stable angina. *Chin. J. Cardiol.* 35 195–206. 10.3760/j.issn:0253-3758.2007.03.002 17582280

[B10] Chu W. C. Wu J. C. Yew D. T. Zhang L. Shi L. Yeung D. K. (2012). Does acupuncture therapy alter activation of neural pathway for pain perception in irritable bowel syndrome?: a comparative study of true and sham acupuncture using functional magnetic resonance imaging. *J. Neurogastroenterol. Motil.* 18 305–316. 10.5056/jnm.2012.18.3.305 22837879 PMC3400819

[B11] Davis K. D. Flor H. Greely H. T. Iannetti G. D. Mackey S. Ploner M. (2017). Brain imaging tests for chronic pain: medical, legal and ethical issues and recommendations. *Nat. Rev. Neurol.* 13 624–638. 10.1038/nrneurol.2017.122 28884750

[B12] Finnerup N. B. Kuner R. Jensen T. S. (2021). Neuropathic Pain: from Mechanisms to Treatment. *Physiol. Rev.* 101 259–301. 10.1152/physrev.00045.2019 32584191

[B13] Fitzcharles M. A. Cohen S. P. Clauw D. J. Littlejohn G. Usui C. Häuser W. (2021). Nociplastic pain: towards an understanding of prevalent pain conditions. *Lancet* 397 2098–2110. 10.1016/s0140-6736(21)00392-5 34062144

[B14] Fraker T. D. Jr. Fihn S. D. 2002 Chronic Stable Angina Writing Committee, American College of Cardiology, American Heart Association, Gibbons R. J. (2007). 2007 chronic angina focused update of the ACC/AHA 2002 guidelines for the management of patients with chronic stable angina: a report of the American College of Cardiology/American Heart Association Task Force on Practice Guidelines Writing Group to develop the focused update of the 2002 guidelines for the management of patients with chronic stable angina. *J. Am. Coll. Cardiol.* 50 2264–2274. 10.1016/j.jacc.2007.08.002 18061078

[B15] Huang S. Li L. Liu J. Li X. Shi Q. Li Y. (2021). The Preventive Value of Acupoint Sensitization for Patients with Stable Angina Pectoris: a Randomized, Double-Blind, Positive-Controlled, Multicentre Trial. *Evid. Based Complement. Alternat. Med.* 2021:7228033. 10.1155/2021/7228033 34765004 PMC8577890

[B16] Jenkinson M. Bannister P. Brady M. Smith S. (2002). Improved optimization for the robust and accurate linear registration and motion correction of brain images. *Neuroimage* 17 825–841. 10.1016/s1053-8119(02)91132-8 12377157

[B17] Lee H. F. Hsieh J. C. Lu C. L. Yeh T. C. Tu C. H. Cheng C. M. (2012). Enhanced affect/cognition-related brain responses during visceral placebo analgesia in irritable bowel syndrome patients. *Pain* 153 1301–1310. 10.1016/j.pain.2012.03.018 22541443

[B18] Liu Q. Zeng X. C. Jiang X. M. Zhou Z. H. Hu X. F. (2019). Altered Brain Functional Hubs and Connectivity Underlie Persistent Somatoform Pain Disorder. *Front. Neurosci.* 13:415. 10.3389/fnins.2019.00415 31114477 PMC6502961

[B19] Ma K. Liu Y. Shao W. Sun J. Li J. Fang X. (2020). Brain Functional Interaction of Acupuncture Effects in Diarrhea-Dominant Irritable Bowel Syndrome. *Front. Neurosci.* 14:608688. 10.3389/fnins.2020.608688 33384580 PMC7770184

[B20] Ma P. Dong X. Qu Y. He Z. Yin T. Cheng S. (2021). A Narrative Review of Neuroimaging Studies in Acupuncture for Migraine. *Pain Res. Manag.* 2021:9460695. 10.1155/2021/9460695 34804268 PMC8598357

[B21] Melzack R. (1975). The McGill Pain Questionnaire: major properties and scoring methods. *Pain* 1 277–299. 10.1016/0304-3959(75)90044-5 1235985

[B22] Mouraux A. Iannetti G. D. (2018). The search for pain biomarkers in the human brain. *Brain* 141 3290–3307. 10.1093/brain/awy281 30462175 PMC6262221

[B23] Piccolo R. Giustino G. Mehran R. Windecker S. (2015). Stable coronary artery disease: revascularisation and invasive strategies. *Lancet* 386 702–713. 10.1016/s0140-6736(15)61220-x 26334162

[B24] Rosen S. D. Paulesu E. Frith C. D. Frackowiak R. S. Davies G. J. Jones T. (1994). Central nervous pathways mediating angina pectoris. *Lancet* 344 147–150. 10.1016/s0140-6736(94)92755-3 7912763

[B25] Saab C. Y. (2012). Pain-related changes in the brain: diagnostic and therapeutic potentials. *Trends Neurosci.* 35 629–637. 10.1016/j.tins.2012.06.002 22763295

[B26] Schwedt T. J. Chiang C. C. Chong C. D. Dodick D. W. (2015). Functional MRI of migraine. *Lancet Neurol.* 14 81–91. 10.1016/s1474-4422(14)70193-0 25496899 PMC11318354

[B27] Shen M. Huang J. Qiu T. (2021). Quality of the Evidence Supporting the Role of Acupuncture for Stable Angina Pectoris: an Umbrella Review of Systematic Reviews. *Front. Cardiovasc. Med.* 8:732144. 10.3389/fcvm.2021.732144 34660732 PMC8514769

[B28] Silvani A. Calandra-Buonaura G. Dampney R. A. Cortelli P. (2016). Brain-heart interactions: physiology and clinical implications. *Philos. Trans. A Math. Phys. Eng. Sci.* 374:20150181. 10.1098/rsta.2015.0181 27044998

[B29] Smith E. R. (2002). The angina grading system of the Canadian Cardiovascular Society. *Can. J. Cardiol.* 18 439–442. 11992140

[B30] Templin C. Hänggi J. Klein C. Topka M. S. Hiestand T. Levinson R. A. (2019). Altered limbic and autonomic processing supports brain-heart axis in Takotsubo syndrome. *Eur. Heart J.* 40 1183–1187. 10.1093/eurheartj/ehz068 30831580 PMC6462306

[B31] Thiebaut de Schotten M. Urbanski M. Valabregue R. Bayle D. J. Volle E. (2014). Subdivision of the occipital lobes: an anatomical and functional MRI connectivity study. *Cortex* 56 121–137. 10.1016/j.cortex.2012.12.007 23312799

[B32] Wei H. L. Li J. Guo X. Zhou G. P. Wang J. J. Chen Y. C. (2021). Functional connectivity of the visual cortex differentiates anxiety comorbidity from episodic migraineurs without aura. *J. Headache Pain* 22:40. 10.1186/s10194-021-01259-x 34020591 PMC8138918

[B33] Wei H. L. Zhou X. Chen Y. C. Yu Y. S. Guo X. Zhou G. P. (2019). Impaired intrinsic functional connectivity between the thalamus and visual cortex in migraine without aura. *J. Headache Pain* 20:116. 10.1186/s10194-019-1065-1 31856703 PMC6924083

[B34] Wen Q. Ma P. Dong X. Sun R. Lan L. Yin T. (2021). Neuroimaging Studies of Acupuncture on Low Back Pain: a Systematic Review. *Front. Neurosci.* 15:730322. 10.3389/fnins.2021.730322 34616275 PMC8488100

[B35] Wittbrodt M. T. Moazzami K. Shah A. J. Lima B. B. Hammadah M. Mehta P. K. (2020). Neural responses during acute mental stress are associated with angina pectoris. *J. Psychosom. Res.* 134:110110. 10.1016/j.jpsychores.2020.110110 32345456 PMC8082434

[B36] Xing J. J. Zeng B. Y. Li J. Zhuang Y. Liang F. R. (2013). Acupuncture point specificity. *Int. Rev. Neurobiol.* 111 49–65. 10.1016/b978-0-12-411545-3.00003-1 24215917

[B37] Yan C. G. Wang X. D. Zuo X. N. Zang Y. F. (2016). DPABI: data Processing & Analysis for (Resting-State) Brain Imaging. *Neuroinformatics* 14 339–351. 10.1007/s12021-016-9299-4 27075850

[B38] Yu S. W. Lin S. H. Tsai C. C. Chaudhuri K. R. Huang Y. C. Chen Y. S. (2019). Acupuncture Effect and Mechanism for Treating Pain in Patients With Parkinson’s Disease. *Front. Neurol.* 10:1114. 10.3389/fneur.2019.01114 31695670 PMC6817566

[B39] Zhao L. Li D. Zheng H. Chang X. Cui J. Wang R. (2019). Acupuncture as Adjunctive Therapy for Chronic Stable Angina: a Randomized Clinical Trial. *JAMA Intern. Med.* 179 1388–1397. 10.1001/jamainternmed.2019.2407 31355870 PMC6664382

[B40] Zhao L. Song Q. Wu H. Wang Y. Wu J. Fang J. (2021). Acupuncture as Adjuvant Therapy for Treating Stable Angina Pectoris with Moderate Coronary Artery Lesions and the Mechanism of Heart-Brain Interactions: a Randomized Controlled Trial Protocol. *Evid. Based Complement. Alternat. Med.* 2021:6634404. 10.1155/2021/6634404 34012473 PMC8105099

