# Supplementary material for: Acupuncture Modulates the Spontaneous Activity and Functional Connectivity of Calcarine in Patients With Chronic Stable Angina Pectoris
Source: Front Mol Neurosci. 2022 Apr 26;15:842674. doi: 10.3389/fnmol.2022.842674 (PMC9087858; doi:10.3389/fnmol.2022.842674)

Supplementary Material

**Table S1. Baseline comparison in fALFF between CSAP patients and HS.**

| **Foci** | **Voxels** | **MNI coordinate** | | | ***t* value** |
| --- | --- | --- | --- | --- | --- |
|  |  | **(x, y, z)** | | |  |
| **Contrast: CSAP > HS** |  |  | | |  |
| Bilateral Calcarine  L Middle occipital gyrus | 573 | 15 | -87 | 21 | 4.619 |
| R Superior temporal gyrus  R Postcentral gyrus | 298 | 57 | -15 | -6 | 3.814 |

Threshold: voxel-level p < 0.05, cluster-level p-GRF < 0.05. Abbreviation: CSAP, chronic stable angina pectoris; HS: healthy subjects, MNI: Montreal Neurological Institute, L: left, R: right.

**Table S2. Acupuncture effects on the fALFF of CSAP patients.**

| **Foci** | **Voxels** | **MNI coordinate** | | | ***t* value** |
| --- | --- | --- | --- | --- | --- |
|  |  | **(x, y, z)** | | |  |
| **Contrast: After treatment > Baseline** | | | | | |
| Brain stem | 210 | -6 | -36 | -57 | 4.826 |
| Brain stem  L Parahippocampus | 107 | -15 | -39 | -12 | 3.762 |
| L Hippocampus | 97 | -18 | -18 | -21 | 3.919 |
| **Contrast: After treatment < Baseline** | | | | | |
| L Calcarine  L Cuneus | 123 | -6 | -69 | 21 | -4.616 |
| R Middle orbitofrontal gyrus | 88 | 36 | 51 | -9 | -4.727 |
| R Median orbitofrontal gyrus | 84 | 6 | 69 | -6 | -3.744 |

Threshold: voxel-level p < 0.05, cluster-level p-GRF < 0.05. Abbreviation: MNI: Montreal Neurological Institute, L: left, R: right.

**Table S3. The between-group difference of fALFF changes after treatment**

| **Foci** | **Voxels** | **MNI coordinate** | | | ***t* value** |
| --- | --- | --- | --- | --- | --- |
|  |  | **(x, y, z)** | | |  |
| **Contrast: (A_pos-_A_pre_) > (B_pos_ - B_pre_)** | | | | | |
| R Cerebellum | 97 | 42 | -54 | -54 | 6.812 |
| **Contrast: (A_pos-_A_pre_) < (B_pos_ - B_pre_)** | | | | | |
| Bilateral thalamus | 139 | 3 | 6 | 15 | -4.957 |

Threshold: voxel-level p < 0.05, cluster-level p-GRF < 0.05. Abbreviation: MNI: Montreal Neurological Institute, R: Right.

**Table S4. The ROI-based FC changes after treatment in group A**

| **Foci** | **Voxels** | **MNI coordinate** | | | ***t* value** |
| --- | --- | --- | --- | --- | --- |
|  |  | **(x, y, z)** | | |  |
| L Cerebellum crus1  L Fusiform gyrus | 225 | -39 | -75 | -24 | 4.627 |
| R Supramarginal gyrus | 176 | 66 | -27 | 42 | 5.230 |

Threshold: voxel-level p < 0.05, cluster-level p-GRF < 0.05. Abbreviation: MNI: Montreal Neurological Institute, R: Right.

**Table S5. The between-group difference of ROI-FC changes after treatment**

| **Foci** | **Voxels** | **MNI coordinate** | | | ***t* value** |
| --- | --- | --- | --- | --- | --- |
|  |  | **(x, y, z)** | | |  |
| L Inferior temporal gyrus  L Cerebellum crus1 | 319 | -45 | -30 | -24 | 4.136 |
| L Hippocampus  L Thalamus | 250 | -39 | -9 | -21 | 4.509 |
| L Middle cingulate cortex | 220 | -27 | -9 | 21 | 5.213 |

Threshold: voxel-level p < 0.05, cluster-level p-GRF < 0.05. Abbreviation: MNI: Montreal Neurological Institute, R: Right.

**Figure S1. The location of the acupoints used in each group**

**
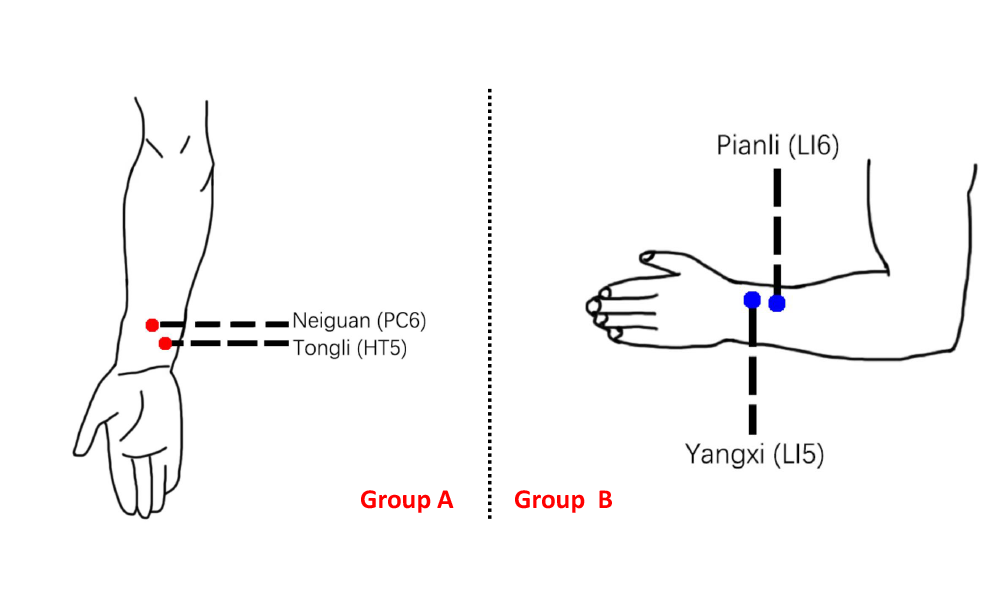
**

Group A (acupoints on the meridian directly related to the Heart): Neiguan (PC6) and Tongli (HT5); Group B (acupoints on the meridian indirectly related to the Heart): Yangxi (LI5) and Pianli (LI6).

**Figure S2. The between-group difference of fALFF changes after treatment**


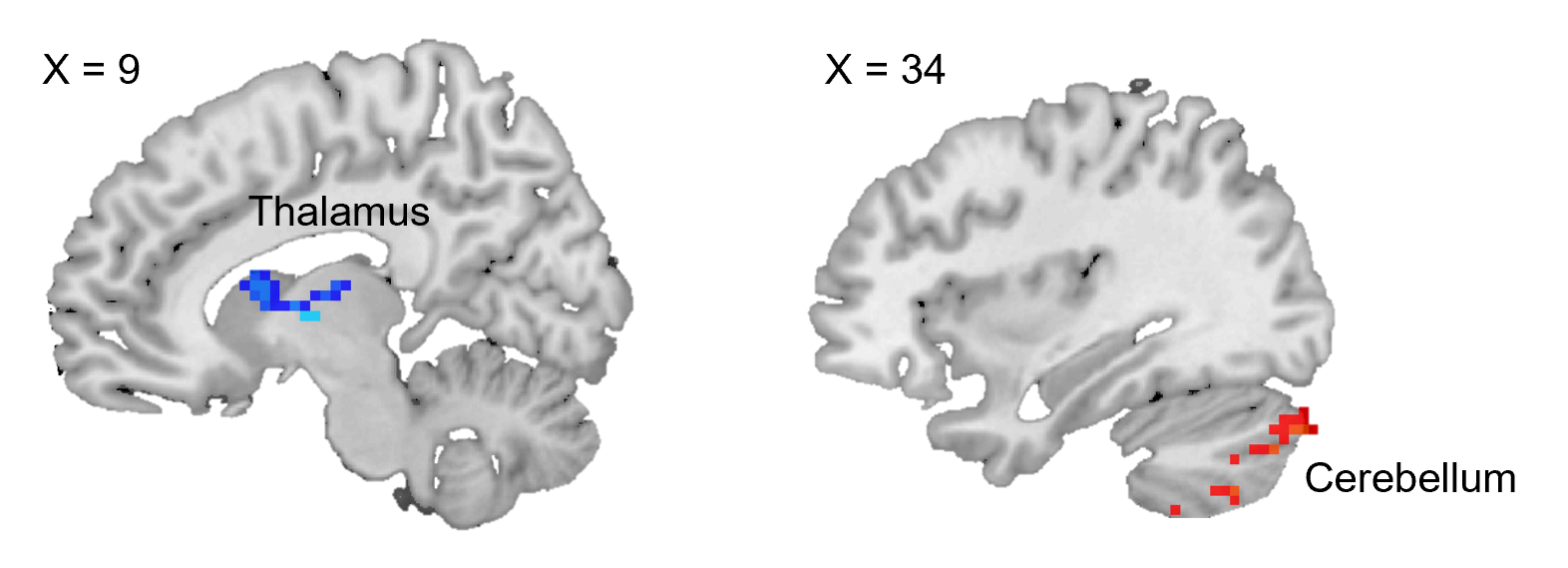

Supplement: Supplementary file 1 [file Data_Sheet_1.docx]
